# Suggestion of suitable animal models for *in vivo* studies of protein tyrosine phosphatase 1b (PTP1B) inhibitors using computational approaches

**DOI:** 10.1186/2193-1801-3-380

**Published:** 2014-07-28

**Authors:** Xuan Thi-Anh Nguyen, Ly Le

**Affiliations:** School of Biotechnology, International University-Vietnam National University, Ward 6, Linh Trung, Thu Duc District Ho Chi Minh City, Vietnam; Life Science Laboratory, Institute for Computational Science and Technology, SBI Building, Quang Trung Software City, Tan Chanh Hiep Ward, District 12, Ho Chi Minh City, Vietnam

**Keywords:** Phylogenetic study, PTP1B, Animal model, Variation, Conservativity, Inhibitor docking

## Abstract

PTP1B is a prototypic enzyme of the superfamily protein tyrosine phosphatases (PTPs) which are critical regulators of tyrosine phosphorylation-dependent signaling events. It is a highly plausible candidate for designing therapeutic inhibitors of obesity and type 2 diabetes (T2D). In this study, a detailed comparative analysis to reveal the evolutionary relationship of human PTP1B among related vertebrates has been addressed.

The phylogenetic trees were constructed with maximum likelihood algorithm by PhyML package on the basis of multiple sequence alignment (MSA) by ClustalΩ and T-coffee. Mutational variability of the sequences corresponding to the 3D structure (pdb: 2vev) was analyzed with Consurf software. The comparative analysis by inhibitor docking to different models was made to confirm the suitability of models.

As a result, the PTP1B or PTP non-receptor type 1 homologies show high conservativity where about 70% positions on primary structures are conserved. Within PTP domain (3–277), the most variable positions are 12, 13, 19 and 24 which is a part of the second aryl binding site. Moreover, there are important evolutional mutations that can change the conformation of the proteins, for instance, hydrophilic N139 changed to hydrophobic Gly (mPTP1B); E132 to proline in the hydrophobic core structure or Y46 to cystein in pTyr recognition loop. These variations/differences should be taken into account for rational inhibitor design and in choosing suitable animal models for drug testing and evaluation. Moreover, our study suggests critically potential models which are *Heterocephalus glaber, Tupaia chinensis, Sus scrofa,* and *Rattus norvegicus* in addition to the best one *Macaca fascicularis.* Among these models, the *H.glaber* and *R.norvegicus* are preferable over *M.musculus* thanks to their similarity in binding affinity and binding modes to investigated PTP1B inhibitors.

**Electronic supplementary material:**

The online version of this article (doi:10.1186/2193-1801-3-380) contains supplementary material, which is available to authorized users.

## Background

Among the PTPs superfamily, PTP1B has become prominent for its down regulation of both insulin and leptin signaling and control of glucose homeostasis and energy expenditure (Tsou and Bence 2012). It terminates the signaling cascade by dephosphorylating the tyrosine residues on its substrates, the phosphotyrosine kinases (PTKs). As a major negative regulator of Janus kinase in JAK-STAT signaling, moreover, PTP1B is recognized to be a key link between metabolic diseases (Tonks 2003), inflammation (Pike et al. 2014) and cancer (Feldhammer et al. 2013).

In insulin signaling, PTP1B acts to dephosphorylate the insulin receptor (IR) at tandem Y1162/Y1163 (Tsou and Bence 2012; Galic et al. 2005) and possibly the insulin receptor substrate 1 (IRS-1) (Galic et al. 2005). Increasing expressed PTP1B and its activity result in over dephosphorylation of IR and kinases leading to interruption of insulin cascades and hence insulin resistance in target tissues.

On the other approach, PTP1B antagonizes leptin signaling via direct dephosphorylation of the active site of the leptin receptor-associated tyrosine kinase JAK2 (Tsou and Bence 2012; Zabolotny et al. 2002; Cheng et al. 2002; Myers et al. 2001). In common obesity, there’s a phenomenon called leptin resistance reflecting the failure of leptin to inhibit energy intake and to increase energy expenditure (Enriori et al. 2006). Since its impact on terminating the leptin signaling, PTP1B is a highly plausible candidate for therapeutic inhibitors to restore leptin sensitivity and prevent disease in the non-adipose tissues (Cook and Unger 2002).

Interestingly, PTP1B-deficient mice were shown to increase insulin sensitivity and resistance to diet-induced obesity (Kahn and Flier 2000; Elchebly et al. 1999; Klaman et al. 2000). Since the discovery of PTP1B in 1988, it has become an important target for treatment of diabetes mellitus and obesity. As over 80% of individuals with T2D are obese (Nadler et al. 2000), PTP1B inhibition may be a potential strategy for a therapeutic target of type 2 diabetes through its links with obesity.

This protein has been well-studied in structure and substrate binding (Tonks 2003). There are four important loops in the catalytic site which are PTP, pTyr, WPD and Q loops. PTP loops contain the signature motif [I/V]HCXXGXXR [S/T] which is highly conserved among classical PTP sub-family. The pTyr loop plays a role in recognition of Tyr tandem in the substrate and contains Tyr46 which defines the depth of the binding site and contributes to absolute substrate specificity of PTP1B to phosphotyrosine-containing substrates. The Cys215 in PTP loop, Asp181 in WPD loop and Gln262 in Q loop are reactive residues essential for catalysis. The second aryl binding site was characterized by Arg24, Arg254 and Gly259 (Andersen et al. 2001). This finding has been supporting variety of PTP1B inhibitor studies (Zhang and Lee 2003).

However, none of potential inhibitors could pass clinical trials which lead to the need of thorough investigating on both functional and evolutionary relationships of PTP1B to other PTPs and among species to avoid inhibitor side effects and to increase suitability of animal *in vivo* test prior to clinical trials. Although the intra-relation among PTP domains of human and vertebrates was reviewed with sequence and partially structure analysis (Andersen et al. 2001), a detailed comparative study to reveal the inter-relation specifically of human PTP1B among related species has not been addressed yet. Hence, the final objective of this study is to propose potentially suitable animal models for *in vivo* drug testing and strategies for further rational inhibitor designs against PTP1B, particularly as treatment for obesity-associated diabetes.

## Results and discussion

### Phylogenetic study of PTP1B protein

**Figure 1:**
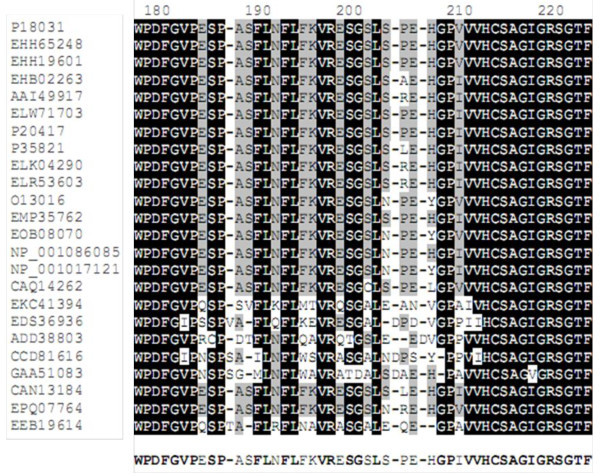
**Multiple sequence alignment (part) of 24 vertebrate PTP1B amino acid sequences.** The consensus sequence obtained with the parameters: identity 91.67%, significance 29.17%, gaps 50%. Residues numbered according to hPTP1B.

The human PTP1B sequence (Uniprot: P18031) was used as template for a protein Blast search of 250 sequences maximum. Selecting from more than 200 sequences, only 27 homologous sequences of PTP1B among different vertebrates qualified for further multiple sequence alignment (MSA) by two algorithms ClustalΩ (Sievers et al. 2011) and T-coffee (Notredame et al. 2000). Comparing the results of the two alignments, there were three more unmatched sequences (GenBank: EFN83906, GenBank: EGW05519, RefSeq: XP_001654306) put aside from the list. The final alignment of 24 homologous sequences was further verified by the algorithm of genetic semihomology (Leluk et al. 2001). The resulting MSA showed relative similarity among sequences. Particularly, the tyrosine-protein phosphatase (PTP) domains (3–277) are well conserved. The PTP signature motif [I/V] HCSAG [I/V] GRS and the WPD-loop motif which are essential for catalysis and substrate trapping, respectively, are completely conserved among the species (Figure 1).

**Figure 2:**
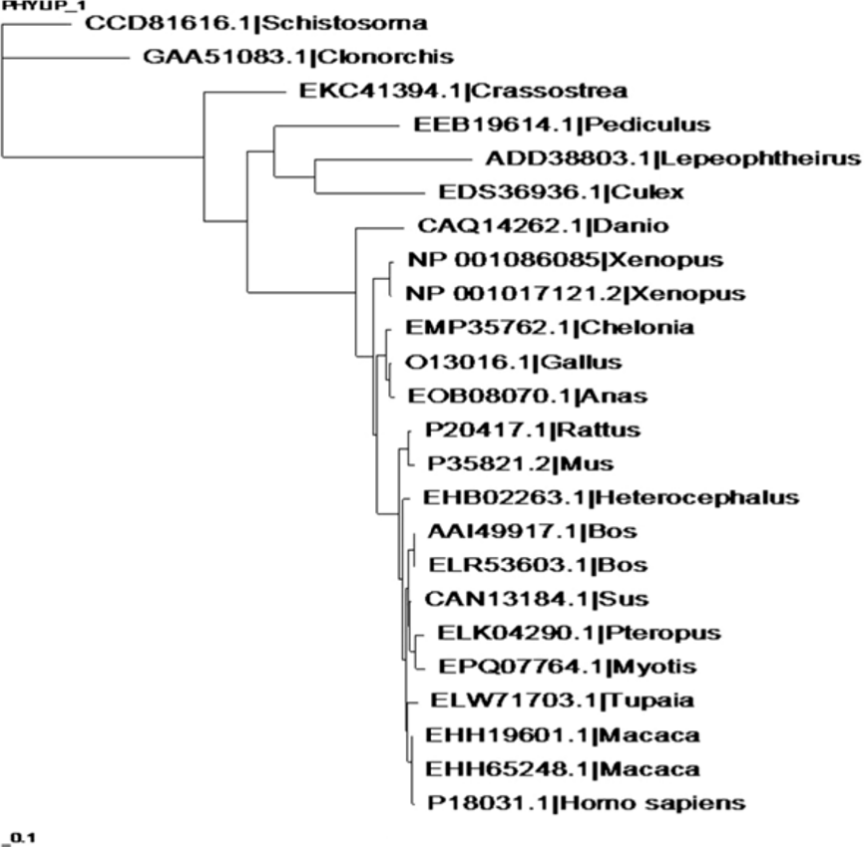
**Unrooted phylogentic tree of 24 species’ PTP1B homologous sequences.** Phylograms obtained by PhyML 3.0.

The refined MSA was used as input for the phylogenetic tree construction by the maximum likelihood algorithm. The resulted phylogram shows two distinct branches (Figure 2). The small group 1 with six distant species including *Schistosoma mansoni, Clonorchis sinensis, Crassostrea gigas, Pediculus humanus corporis* and *Culex quinquefasciatus.* The larger group 2 with 17 species starts from *Danio* to *Homo sapiens.* Group 2 can also be divided into 3 subgroups (aside from *Danio*) which are *Xenopus* group (subgroup 1); Chelonia and poultry species (subgroup 2); and the biggest subgroup 3 ranging from rodent species to human.

**Figure 3:**
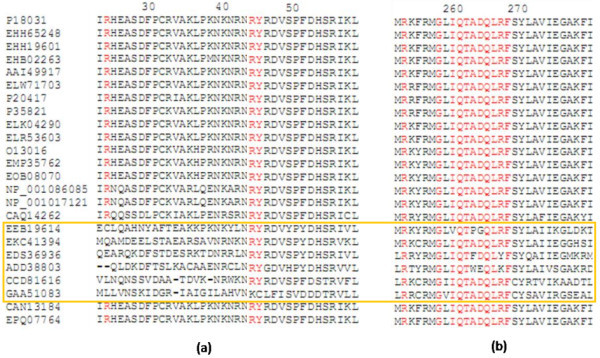
**Variations in important binding sites of some sequences – (a) R24 second aryl binding site and pTyr recognition site; (b) R254 & G259 second aryl binding site and Q-loop motif.** Conserved residues in these positions are shown in red. The yellow square indicates 6 species that have vigorous variations in these regions.

Protein sequences from monkey species *Macaca fascicularis*, *Macaca mulatta* have the closest vicinity to hPTP1B. However, they might not be preferable as animal models because of bioethics for drug test in some cases. The next important candidate is the Chinese treeshew *Tupaia chinensis.* Although the sequence cover is not closely guaranteed as *Tupaia*’s sequence is longer (598aa) than that of human and therefore could lead to disagreement in protein structure, the amino acid identity is high in critical positions (refer to Figures 1 and 3).

Essentially, hPTP1B (P18031), in this study, acts as indicator for choosing suitable animal models for in vivo tests due to its relevance to clinical studies for drug targeting (Sobhia et al. 2012). For this reason, group 1 was not chosen for further analysis because of distant evolution from hPTP1B. Furthermore, ptp1b sequences from these species reveal critical variations/mutations in PTP domains (Figure 3). Arg45 and Tyr46 in pTyr recognition loop are mutated to Lys and Cys respectively in *Clonorchis*. Within the Q loop (262–269), there are variations observed in *Pediculus* (I-V; A-P; D-G), *Culex* (A-F; R-Y), *Lepeophtheirus* (A-W; R-K). Among those, the mutations from Asp265 (negatively charged) to Gly (hydrophobic) in *Pediculus* may affect the conformation of the loop. Looking into the second aryl binding site of the protein (Andersen et al. 2001), Arg24 is quite varied in group 1 sequences. Point mutations from R (positively charged) to E (negatively charged), to L (hydrophobic) or even deleted (gapped) may cause significant differences in substrate trapping/interaction of the PTP1B in these species from that of hPTP1B.

### Analysis on evolutionary conservation

The PTP1B homologous sequences of group 2 among 18 selected species including human were analyzed thoroughly by Consurf server. This test not only helped resolve which are the most variable/conserved regions on the protein but also contributed to the selection of proper animal models.

**Figure 4:**
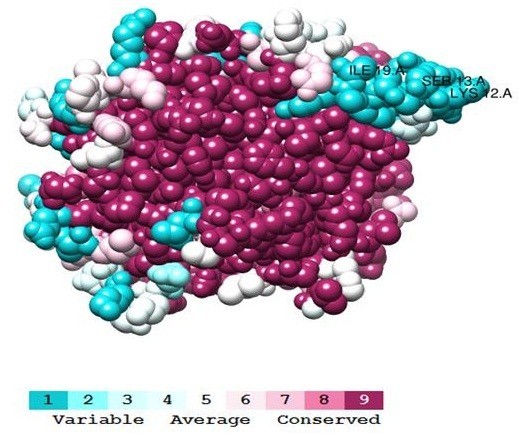
**Mutational variability of 18 aligned PTPN1 sequences in corresponding to PTP1B structure [PDB: 2VEV].** Labeled residues indicate the most variable region(s). The figure was prepared by Chimera 1.8 with Consurf color codes.

Overall, the PTP1B protein is highly conserved at the core structure of the catalytic domain (pdb: 2vev). There are 219 positions absolutely conserved through evolution. Forty-eight positions are indicated with 2 different residues while 27 positions with 3 various residues. A variety of 4 residues occurs in 14 positions and 6 positions reveal high variations of 5 or 6 residues. The most varied positions are 12, 13 and 19 which in hPTP1B are lysine, serine and isoleucine (Figure 4).

**Figure 5:**
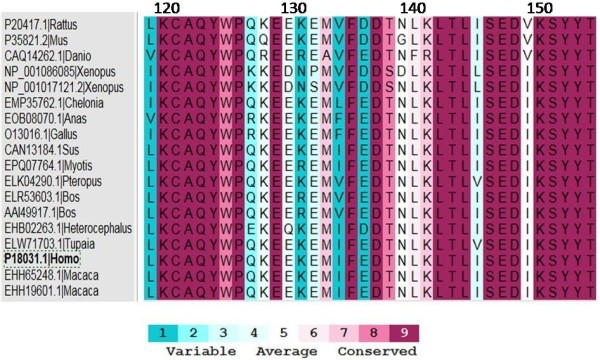
**Consurf color-coded multiple sequence alignment (part) with conservativity score of 18 PTP1B homologous sequences.**

Particularly, the variable residues/regions adjacent to the conserved motifs range from 1 to 5 levels according to Consurf color-coded MSA (Figure 5). Within the motif KCAQYWP, the hydrophobic core structure, that interacts with ligand induced residues (Andersen et al. 2001), for instance, E132 in hPTP1B with a negatively charge is exceedingly different from Pro in *Xenopus laevis*. Another example is hydrophilic N139 variated significantly into hydrophobic Gly in mPTP1B of *Mus musculus* cautioning that this rodent might not be a good model for PTP1B inhibitor-related studies. These mutations can cause differences in conformation between the proteins of human and the various species.

Based on the percentage of variations among sequences to hPTP1B (data not shown) and the level of mutations as well as the phylogenetic information, a table ranking potential animal models was formed for later references (Table 1). Noticeably, species of the subgroup 3 in branch 2 of the phylogenetic tree were among top of the rank and hence considered as subjects for further analysis. Because of bioethic issues related to primate species and economic issues of *Bos* genus, however, there are only 7 models suggested for next comparative analysis with inhibitor dockings.

**Table 1:** **Ranking the candidates based on variation/conservativity level within PTP domains (275 residues)**

Ranking	Species	Accession number
1	1.1.*Macaca fascicularis* (*long-tailed macaque)*	1. EHH65248
	1.2.*Macaca mulatta* (*rhesus monkey)*	2. EHH19601
2	*Bos taurus* (domestic cow)	AAI49917
3	*Sus scrofa* (wild pig)	CAN13184
4	*Heterocephalus glaber* (*naked mole rat)*	EHB02263
5	*Tupaia chinensis* (tree shrew)	ELW71703
6	6.1.*Pteropus alecto* (black flying-fox)	6.1. ELK04290
	6.2.*Myotis brandtii* (brandt’s bat)	6.2. EPQ07764
7	*Bos grunniensmutus* (wild yak)	ELR53603
8	*Rattus norvegicus* (rat)	P20417.1
9	*Mus musculus* (mouse)	P35821.2
10	*Gallus gallus* (chicken)	O13016
11	*Chelonia mydas* (green turtle)	EMP35762
12	*Anas platyrhynchos* (wild duck)	EOB08070
13	*Xenopus (Silurana) tropicalis* (tropical clawed frog)	NP_001017121
14	*Xenopus laevis* (African clawed frog)	NP_001086085
15	*Danio rerio* (zebrafish)	CAQ14262

### Inhibitor docking into models’ PTP1B 3D structures

Seven candidates, *Sus scrofa*, *Tupaia chinensis*, *Heterocephalus glaber, Myotis brantdii, Pteropus alecto, Rattus norvegicus* and *Mus musculus,* which are available and have high potential were chosen for further analysis on structures and ligand interactions. The PTP1B proteins of these animals have no experimental structures yet; hence they are modeled as homologs from the template 2VEV of human PTP1B catalytic domain with 299 residues. The sequence identity of models to human template is over 80% and the overall pattern of the structure of PTP1B catalytic domain is conserved (Additional file 1).

These models, along with hPTP1B (pdb: 2vev), were investigated as to their ligand interaction by inhibitor docking with Ertiprotafib (K_i_ 1500 nM) and five other small molecules published as potential PTP1B antagonists denoted as compounds 1 to 5 (Zhang and Lee 2003). Compound 1 (affinity 220 nM) is peptidomimetics of 3-carboxy-4-(O-carboxymethyl) tyrosine core that could augment insulin action in the cell (Larsen et al. 2003). Compound 2 (K_i_ 2 μM) is the ortho tetrazole analogue in which tetrazole moiety is well-accommodated in the active site (Liljebris et al. 2002). Compound 3 (K_i_ 0.6 μM) was developed by Novo Nordisk group to address the second aryl phosphate-binding pocket of PTP1B (Iversen et al. 2001). Abbott group investigated about compound 4 (K_i_ 77 nM) for interacting with both binding sites on the PTP1B enzyme (Liu and Trevillyan 2002). The non-hydrolyzable analog, compound 5 (K_i_ 2.4 nM), was the most potent inhibitor for being capable of occupying both active site and a unique peripheral site (Shen et al. 2001).

**Figure 6:**
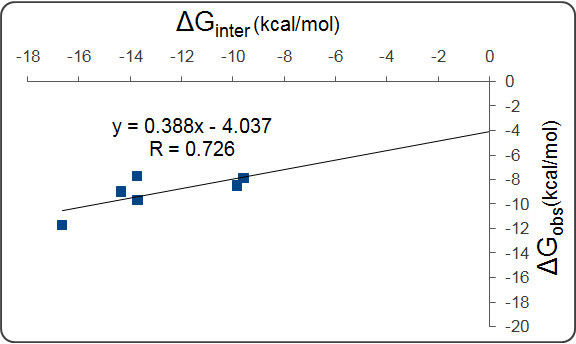
**The correlation between the computational interaction energies and the observed binding energies (** **ΔG**_**obs**_ **) calculated from the experimental K**_**i**_ **values of investigated inhibitors.** ΔG_obs_ = *RT* lnK_*i*_ with ΔG_obs_: *observed free energy change of binding*; K_*i*_ *: inhibition constant*; *R: gas constant (1.987 cal K* ^*-1*^ *mol* ^*-1*^ *)*; *T: room temperature (298.15 K)*.

Because most of these inhibitors have a high number of torsions, the docking scores estimated by AutoDock Vina were calculated into the binding energies without torsion interferences (Table 2). These computational ΔG_inter_ values were in relatively strong correlation (Figure 6) with the observed binding energies calculated from the experimental K_i_ values.

**Table 2:** **Calculated binding energies** **ΔG**_**inter**_ **(kcal/mol) of six PTP1B inhibitors to protein models with hPTP1B as standard**

	H. sapiens	S. scrofa	T. chinensis	H. glaber	M. brandtii	P. alecto	R. norvegicus	M. musculus
Ertiprotafib	-9.6	-9.6	-10.8	-9.6	-9.7	-9.7	-9.7	-9.6
Compound 1	-14.3	-13.5	-15.4	-14.6	-13.7	-13.7	-13.5	-13.7
Compound 2	-13.7	-14.8	-15.4	-14.2	-14.2	-13.7	-14.8	-13.9
Compound 3	-9.8	-9.8	-10.3	-9.8	-8.2	-9.7	-9.7	-9.5
Compound 4	-13.7	-13.9	-16.7	-14.2	-12.8	-13	-13.7	-12.3
Compound 5	-16.7	-14.1	-17	-13.9	-14.1	-15	-17.3	-14

**Figure 7:**
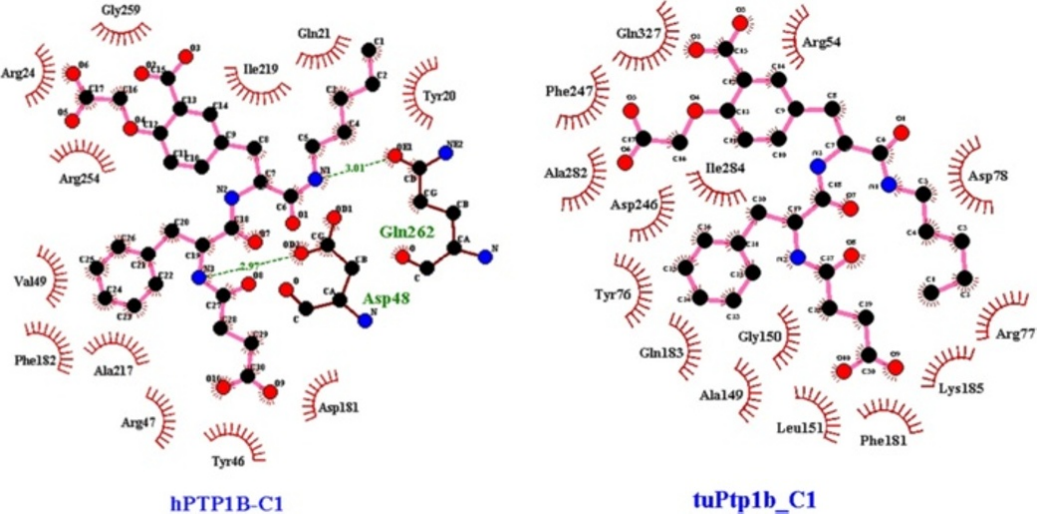
**Comparison in the binding site of hPTP1B (left) and of TuPTP1B (right) to the peptidomimetic compound 1.** The binding pockets are visualizaed by LigPlot^+^ v.1.4. The ligands and protein side chains are shown in ball-and-stick representation, with the ligand bonds coloured in pink. Hydrogen bonds are shown as green dotted lines with H-bond lengths. Residues with direct/hydrophilic contacts are colored in green with brown backbone whereas ones with indirect/hydrophobic interactions are colored in black and indicated with the red spoked arcs.

**Figure 8:**
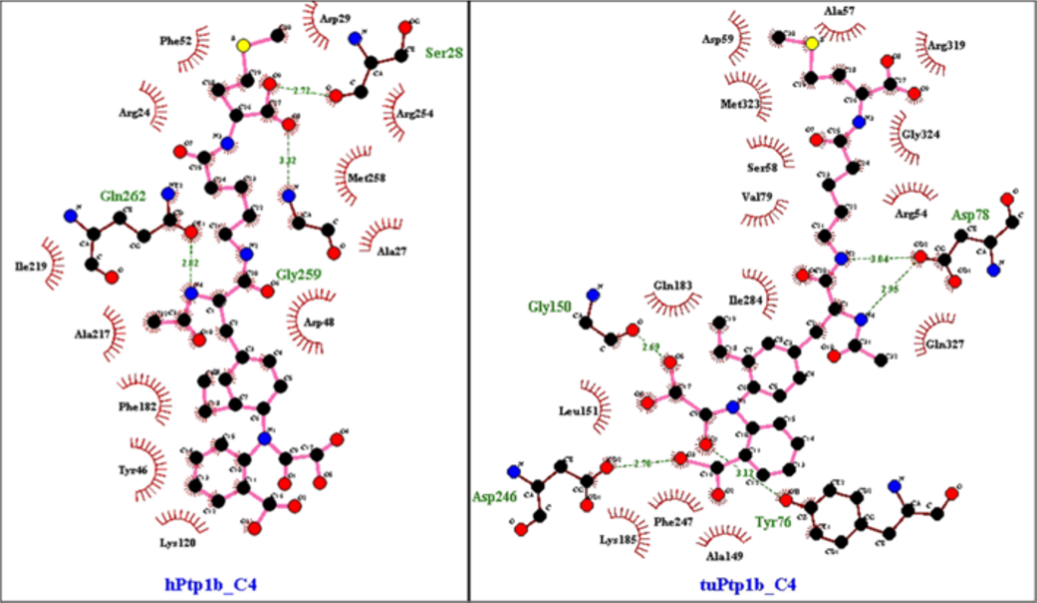
**Differences in binding sites of hPTP1B (left) and** ***T.chinensis*** **PTP1B (right) to compound 4.** The analysis and illustration were made by using LigPlot^+^ v.1.4.

**Figure 9:**
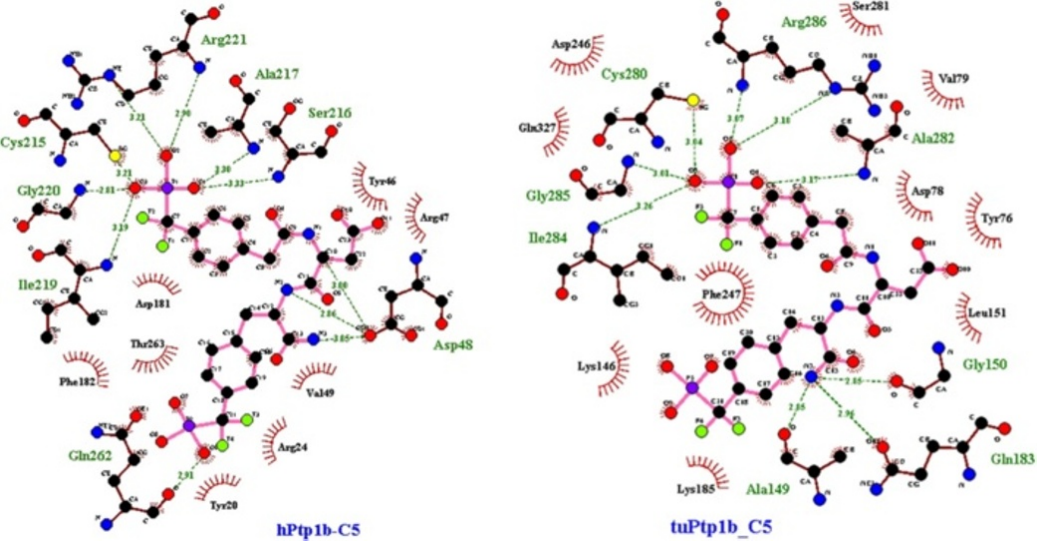
**Comparison of the binding pocket of hPTP1B (left) and** ***Tupaia*** **PTP1B (right) for compound 5.** The analysis and illustration were made by using LigPlot^+^ v.1.4.

*T. chinensis* model had the strongest affinity to all six inhibitors but showed differences with hPTP1B in binding modes of compound 1, 4 and 5. The binding site of TuPTP1B did not have direct contact at residues Gln262 and Asp48 with compound 1 as the hPTP1B or mole-rat PTP1B had (Figure 7). In the case of compound 4, hydrophilic interaction of this ligand involved the residues Gln262, Gly259 and Ser28 in hPTP1B whereas it happened around carboxylic group of the aromatic moiety with tyrosine, glycine and aspartate residues in *Tu*PTP1B (Figure 8). *Tupaia* PTP1B also did not form hydrogen bonds with compound 5 at residues Ser216 and Asp48 as in hPTP1B; instead it had indirect contact with these residues (Figure 9).

**Figure 10:**
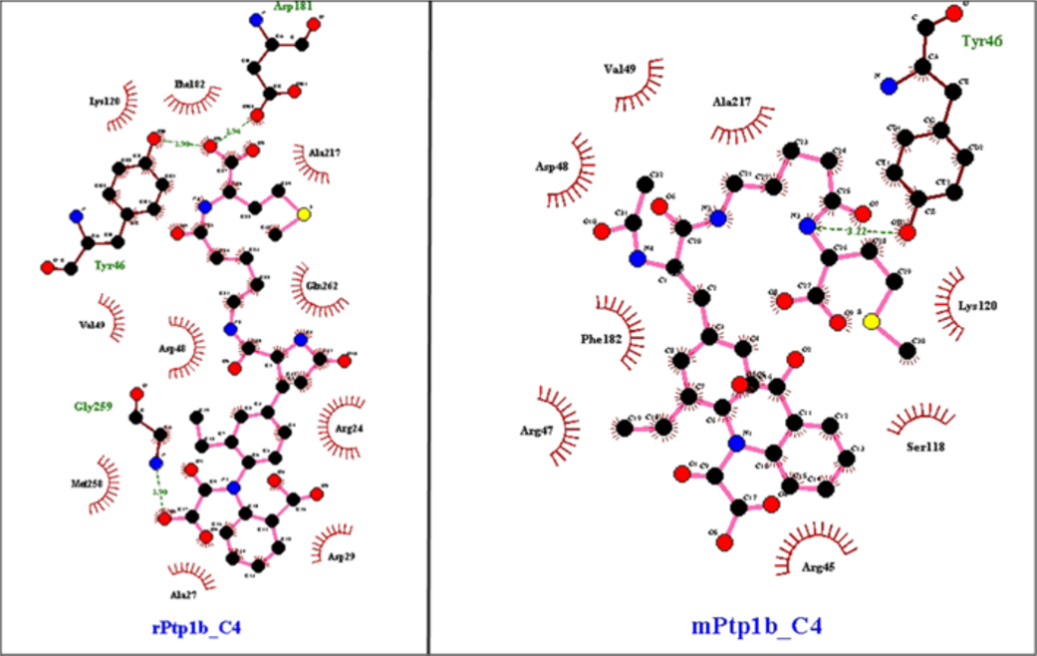
**Similarity of binding pocket of rat PTP1B model to hPTP1B leading to superiority of rat model over the mouse model – specific case with compound 4.** The analysis and illustration were made by using LigPlot^+^ v.1.4.

**Figure 11:**
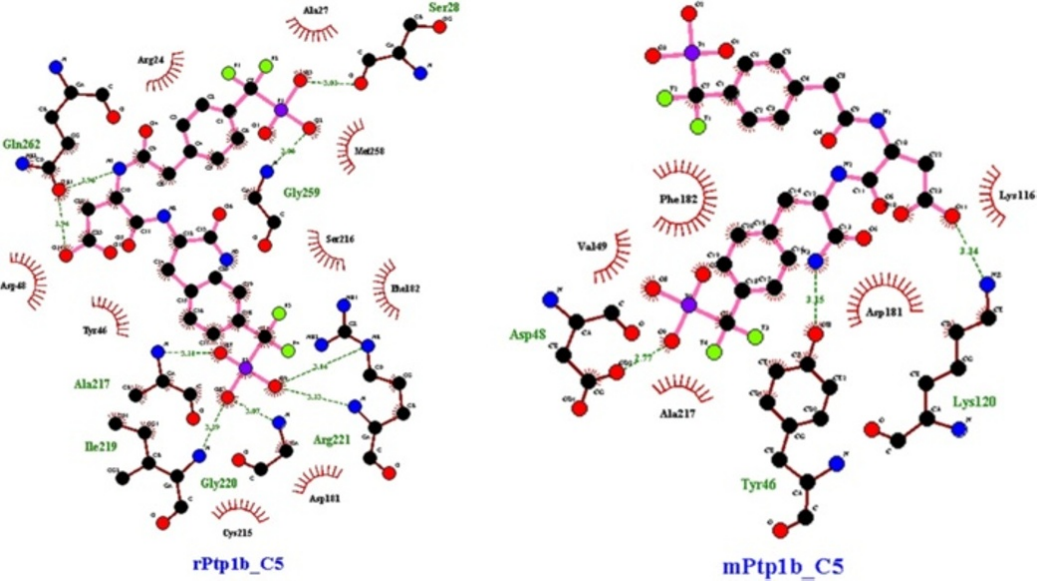
**Similarity of the binding pocket of rat PTP1B model to hPTP1B leading to superiority of rat model over the mouse model – specific case with compound 5.** The analysis and illustration were made by using LigPlot^+^ v.1.4.

Noticeably, the *R.norvegicus* (rat) and *H.glaber* (mole-rat) models appeared to be best suited for inhibitor studies of hPTP1B. Most of the inhibitors docked into these models have close docking scores and rather similar binding modes with those in hPTP1B except for compound 1 and compound 5 respectively (data not shown). In this test, the *M.musculus* PTP1B was again recognized to be less preferable than the rat particularly with compound 4 and 5. While rPTP1B maintained the direct contact with Gly259 to O8 of compound 4 and most of other indirect contacts, mPTP1B revealed significant difference as it solely had H-bonding to the ligand at Tyr46 in the active site (Figure 10). Especially, mPTP1B has less affinity to compound 5 and a different binding site for this molecule than hPTP1B whereas rPTP1B showed the most similarity. There are at least five H-bonds formed between Gln262, Ile219, Gly220, Arg221, Ala217 of rPTP1B and inhibitor 5 as in hPTP1B binding pocket but mPTP1B could only preserve the contact of Asp48 with the molecule (Figure 11).

*P.alecto* had strong affinity to compound 2, just as hPTP1B does but it had weak affinity to compound 4 and 5. Most models had relatively good binding affinity to compound 3, particularly the *S.scrofa* and *H.glaber* models responsed the same as hPTP1B. However, in this study, the hPTP1B binding site for compound 3 showed slight differences to the experimental report (Iversen et al. 2001). We could not observe the salt bridge between the molecule and Asp48 because, in our study, there was no water molecule introduced during the conventional docking procedure.

## Conclusions

This study intensively analyzes the phylogenetic relationship between hPTP1B and other common vertebrates. Important mutations/variations in second-aryl binding sites, adjacent regions of Q loop and hydrophobic core structure should be noticeable as protein conformational differences which are likely to lead to disagreement between *in silico* design and in vivo testing. Rats, as a common model, are more preferable for having higher similarity with hPTP1B than mice while *Heterocephalus glaber* emerges as new model due to better suitability and agreement in the target PTP1B sequences.

Among all, *H.glaber* and *R.norvegicus* are preferred over *M.musculus* thanks to their similarity in binding affinity and binding modes to investigated PTP1B inhibitors. They are also more common and available than other animals as models for *in vivo* tests.

It is recommended that the study can be scaled up for investigating more variety of potential PTP1B inhibitors in these animal models. It is also necessary to study whether functions of PTP1B homologs in these animals are similar in human or not. In order to ensure the success of drug development as well as to reduce time and cost, the suitability of animal tests is very critical to prevent false positive results.

## Methods

### Multiple sequence alignment and phylogenetic tree construction

Full-length sequence of hPTP1B with 435 amino acids (Swiss-Prot: P18031) collected from the UniProtKB database (http://www.uniprot.org/) was the query sequence for a Blastp (Altschul et al. 1990) search from the non-redundant protein database with default parameters (BLOSUM 62 matrix (Henikoff and Henikoff 1992)). From a maximum 250 homologies, qualified sequences which could represent the PTP1B homologs in different vertebrates were the materials for multiple sequence alignment (MSA) using Clustal Omega (Sievers et al. 2011) with input ordered and Phylip output format (http://www.ebi.ac.uk/Tools/msa/clustalo/). The result of MSA was also compared and verified by T-Coffee method (Notredame et al. 2000) and the algorithm of genetic semihomology (Leluk 1998; Leluk et al. 2001) respectively. The consensus sequence of aligned PTP-non receptor type 1 sequences was then constructed with the aid of Consensus constructor (Fogtman and Lesyng 2005). The parameters used were: identity 91.67%, significance 29.17%, gaps 50%. The refined MSA was then used as input for the construction of a phylogenetic tree by the PHYML approach (Guindon and Gascuel 2003) which implements the maximum likelihood method. The options were adjusted for amino acid data type, Jones, Taylor, and Thornton (JTT) substitution model and tree topology best searching of NNI (Nearest Neighbor Interchange) and SPR (Subtree Prune and Regraft) search.

### Analysis of evolutionary conservativity

The evolutionary conservativity/variability of aligned protein homologies was calculated with the help of Consurf (Glaser et al. 2003; Landau et al. 2005; Ashkenazy et al. 2010) (http://consurf.tau.ac.il/). The conservativity scores were calculated by Bayesian method. JTT was the evolutionary substitution model applied. Evolutionarily functional positions and regions were also analyzed on the basis of the hPTP1B structure [PDB: 2VEV] and visualized by Chimera (Meng et al. 2006) version 1.8 (http://www.cgl.ucsf.edu/chimera/).

### Inhibitor docking into PTP1B models

The 3D structures of PTP1B of most potential animal candidate were constructed by homology modeling on the Swiss-Model server (http://swissmodel.expasy.org) from the template hPTP1B catalytic domain structure (residues 1–321) on Protein Data Bank (PDB: 2VEV). The model quality was mainly evaluated based on the QMEAN4 score which is a composite score consisting of a linear combination of 4 statistical potential terms (estimated model reliability between 0–1) and RMSD values.

Six PTP1B inhibitors reviewed (Zhang and Lee 2003) were prepared in 3D structures. The docking step between newly modeled protein structures and these inhibitors was undergone by AutoDock Vina package (Trott and Olson 2010). The ligands were prepared by the graphical user interface AutoDockTools (http://mgltools.scripps.edu/downloads). The input ligands were added Gasteiger charged if missing, merged non-polar H, detected rotatable bonds and then set Torsion degree of freedom (TORSDOF). The receptors were also prepared as pdbqt file with the grid map information. The center of the grid box was (17, 18, 77) and applied to all the receptor structures as they are written in the same pattern of coordinates. This box has the size of 30Ǻ at each square face and cover both known binding pockets of PTP1B. The docking step was run with two CPU, exhaustiveness 10 and only the binding mode with the lowest free binding energy was recorded. The resulting docking scores were the predicted free binding energies (Gibbs, ΔG) with the intramolecular contributions taken into account (c = c_inter_ + c_intra_). The predicted docking scores in this study were then re-calculated into the interaction energies that avoid the interferences caused by high torsion numbers of the inhibitors (with more than 10 rotatable bonds). The following formula helped compute the final binding energies:

ΔG_inter_ = ΔG_pred_ *(1 + 0.05846 N_rot_) (Trott and Olson 2010)

Binding modes were further analyzed in the context with protein binding sites by LigPlot^+^ v.1.4 (Laskowski and Swindells 2011).

## Electronic supplementary material

Additional file 1: **Quality of PTP1B models built by homology modeling on Swiss-Model server (** http://swissmodel.expasy.org **) with hPTP1B (pdb: 2vev) as template.** (PDF 180 KB)
