# Supplementary material for: Suggestion of suitable animal models for in vivo studies of protein tyrosine phosphatase 1b (PTP1B) inhibitors using computational approaches
Source: Springerplus. 2014 Jul 28;3:380. doi: 10.1186/2193-1801-3-380 (PMC4132456; doi:10.1186/2193-1801-3-380)
Supplement: Supplementary file 1 — Additional file 1: Quality of PTP1B models built by homology modeling on Swiss-Model server ( http://swissmodel.expasy.org ) with hPTP1B (pdb: 2vev) as template. (PDF 180 KB) [file 40064_2014_1115_MOESM1_ESM.pdf]

Additional file. Quality of PTP1B models built by homology modeling on Swiss-Model server (http://swissmodel.expasy.org) with hPTP1B (pdb: 2vev) as template

Species	No.of Residues	Sequence Identity (%)	QMEAN4 score	RMSD (Å)	3D structure
H.sapiens (pdb: 2vev)	299	100%	1	0	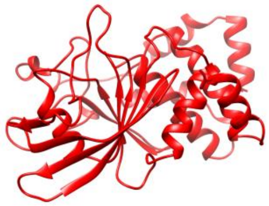
H.glaber	298	96.98	0.757	0.063	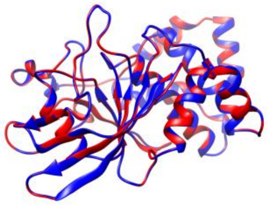
M.musculus	298	94.966	0.764	0.063	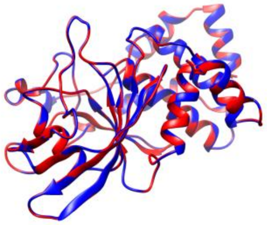
M.brandtii	279	97.849	0.747	0.062	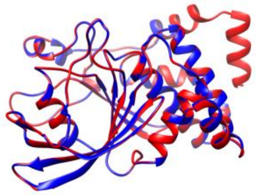
S.scrofa	298	97.987	0.767	0.063	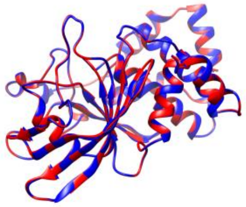

P.alecto	278	97.122	0.758	0.062	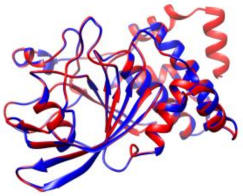
R.norvegicus	298	96.309	0.766	0.065	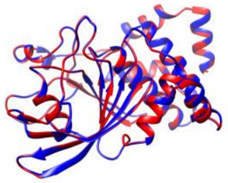
T.chinensis	363	80.716	0.644	0.099	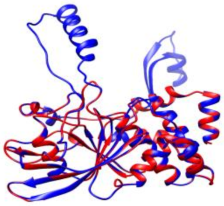
